# Exploring the association between the Arabic version of the Traffic Locus of Control, driving behavior, and road traffic crashes: A multidimensional approach

**DOI:** 10.1371/journal.pone.0303518

**Published:** 2024-05-23

**Authors:** Dalal Youssef, Pascale Salameh, Louis-Rachid Salmi

**Affiliations:** 1 Institut de Santé Publique d’Epidemiologie et de Development, Bordeaux University, France, UMR_S 1219—Research Center Bordeaux Population Health (BPH), Bordeaux, France; 2 Clinical Trial Program, Ministry of Public Health, Beirut, Lebanon; 3 Lebanese Higher Institute of Technical & Professional (IPNET), Beirut, Lebanon; 4 Institut National de Santé Publique, Epidémiologie Clinique et Toxicologie (INSPECT-LB), Beirut, Lebanon; 5 School of Medicine, Lebanese American University, Byblos, Lebanon; 6 Department of Research, Faculty of Pharmacy, Lebanese University, Hadat, Lebanon; 7 Department of Primary Care and Population Health, University of Nicosia Medical School, Nicosia, Cyprus; AUM: American University of the Middle East, KUWAIT

## Abstract

The Traffic Locus of Control scale (T-LOC) serves as a measure of drivers’ personality attributes, providing insights into their perceptions of potential causes of road traffic crashes (RTCs). This study meticulously evaluated the psychometric properties of the Arabic version of T-LOC (T-LOC-A) among Lebanese drivers. Additionally, the study aimed to explore associations between the T-LOC scale and various driving variables, including driver behavior, accident involvement, and traffic offenses. A cross-sectional study was conducted among Lebanese drivers using a face-to-face approach. The validation of the Arabic version of T-LOC (T-LOC-A) occurred through a two-stage process: translating and culturally adapting T-LOC in the first stage, and testing its psychometric properties in the second stage. Data were collected using a comprehensive self-reported questionnaire in Arabic, covering demographic and travel-related variables, risk involvement, and measures such as the Driver Behavior Questionnaire (DBQ) and T-LOC. Exploratory factor analysis and confirmatory factor analysis were performed to scrutinize the factorial structure of T-LOC. Pearson correlation and chi-square tests were used for continuous and categorical variables, respectively. Two logistic regression analyses were executed to probe associations between T-LOC and involvement in road traffic crashes (RTCs) and T-LOC subscales with the occurrence of traffic offenses. The study included 568 drivers, predominantly male (69%) and aged between 30 and 49 years (42.1%). The findings revealed that T-LOC-A exhibited robust psychometric properties, with excellent reliabilities (α = 0.85) and adherence to the original four-factor structure, encompassing self (α = 0.88), other drivers (α = 0.91), vehicle/environment (α = 0.86), and fate (α = 0.66). The multidimensional structure was statistically supported by favorable fit indices. Gender differences revealed men attributing responsibility to other drivers, while women leaned towards fate and luck beliefs. Regarding driver behavior, the "other drivers" and self-dimensions of T-LOC-A correlated positively with aggressive violations. The fate dimension showed positive associations with aggressive violations and lapses. The "other drivers" subscale correlated positively with errors, and the vehicle/environment subscale with lapses. External T-LOC factors were positively associated with accident involvement, while the "LOC self" factor emerged as a protective element. In terms of traffic offenses, "LOC fate" displayed a positive association, while the "LOC self" factor showed a protective effect. In conclusion, the Arabic T-LOC is a reliable and valuable instrument, suggesting potential improvements in driving safety by addressing drivers’ locus of control perceptions.

## I. Introduction

Globally, road traffic crashes (RTCs) stand as a prominent contributor to injury-related fatalities. Recognizing the human factor as a pivotal determinant in RTCs [1], a thorough examination becomes imperative for decision-makers. This scrutiny not only aids in the identification of key factors but also facilitates the formulation of impactful road safety interventions. Consequently, the development of strategies to enhance the safe operation of motor vehicles emerges as a crucial avenue, promising substantial societal and organizational impact.

### Locus of control

The concept of Locus of Control (LOC), developed by Rotter, reflected an individual’s habitual cognitive processing style and belief about the consequences of actions [2]. People generally perceive situations to be under their control or under external forces. Since the introduction of this concept, it has garnered extensive popularity and application across various research domains, spanning from health psychology to traffic safety. External-LOC personalities perceive outcomes as the consequence of external, uncontrollable influences like luck, fate, and powerful others, whereas internal-LOC personalities link outcomes to their behavior. The concept of LOC illuminates individual disparities in perceiving the causal connection between actions and outcomes [3]. This means that individuals with different levels of locus of control (LOC) may possess diverse perceptions of their ability to control events and outcomes in their lives.

### Traffic locus of control

Within the realm of traffic safety, researchers acknowledge the locus of control (LOC) as a pivotal facet of an individual’s personality capable of shaping their perception of control over events [2]. Consequently, this construct assumes a significant role in forecasting safe driving practices and discerning drivers’ risky behaviors and involvement in crashes [4–7]. Therefore, Özkan & Lajunen (2005) [8] developed the multidimensional Traffic Locus of Control scale (T-LOC), which is a widely used tool for assessing the LOC of drivers. Comprising 15 items, T-LOC measures an individual’s LOC orientation across four factors that encompass all potential causes of traffic accidents. These factors are "self", "other drivers", "vehicle/environment", and "fate". “Self” represents drivers’ internal beliefs, and the other three factors represent external factors [8]. Since its development, the T-LOC scale has garnered significant attention from researchers and has been translated into several languages to facilitate cross-cultural research. The Swedish [9] and Romanian [10] versions did not conserve the same factorial structure, therefore suggesting that the factorial structure of the T-LOC must be adapted for use in other cultural contexts and driving environments. Hence, a further component was added to the original T-LOC in both of the mentioned versions. In regard to the Swedish version, it comprises 17 items, and the component called “self” in the original version T-LOC was split into two factors known as “own skills” and “own behavior” [9]. In the Romanian version, religiosity was added as a new component and the scale was extended to include 41 items [10]. Despite the modifications between versions, the principal four factors in the original T-LOC were kept. This highlights the importance of adaptation of the instrument according to the culture.

### T-LOC and driver behavior

The impact of T-LOC on traffic safety has prompted researchers to investigate its relationship with various driving behaviors [8–10]. According to Montag and Comrey’s (1987) study [6], drivers who attributed the cause of road traffic collisions (RTCs) to external LOC factors were more likely to engage in risky driving behaviors [7]. Conversely, drivers attributing driving outcomes to internal and controllable factors [11] are more motivated to adopt safe-driving behavior because these individuals were more likely to adopt defensive driving and precautionary measures [12, 13]. For instance, individuals with high internal LOC scores tend to exhibit responsible driving behaviors, including consistent seatbelt use [13, 14], heightened alertness while driving [15] and prompt application of brakes when anticipating potential dangers on the road. This recognition is supported by numerous empirical studies demonstrating a clear association between LOC and various behaviors pertinent to traffic safety [16, 17]. These findings underscore the importance of understanding an individual’s T-LOC orientation in promoting responsible driving habits and reducing the risk of accidents on the road.

### Lebanese context

In Lebanon, road traffic crashes (RTCs) pose a significant threat to community health, exacerbating the strain on an already overwhelmed healthcare sector. The dearth of data and the limited analysis of factors contributing to road traffic injuries (RTIs), encompassing human, vehicle, and environmental elements and their interactions, hampers the implementation of targeted interventions. Recognizing that driver behavior is a pivotal factor, exploring the personality traits influencing drivers and their potential contribution to RTCs becomes essential for effective human behavior interventions.

Given previous research indicating the significance of Traffic Locus of Control (T-LOC) in predicting risk-taking behaviors and traffic accidents among drivers, particularly those with external orientations [18, 19], there is a compelling need to investigate this attribute among Lebanese drivers using a validated scale. In addition, the absence of prior investigations into the role of Locus of Control (LOC) among Lebanese drivers underscores the necessity for a reliable tool to evaluate drivers’ LOC. However, before embarking on this study, it is imperative to validate the T-LOC scale among Lebanese drivers. This validation ensures its reliability in capturing drivers’ perceptions of control within the distinctive cultural context, laying the groundwork for accurate assessments of psychological factors influencing driving behavior and RTCs. Furthermore, the validated T-LOC scale not only supports targeted interventions for enhancing road safety in Lebanon but also contributes valuable insights to global traffic psychology knowledge, fostering cross-cultural research and facilitating meaningful comparisons between countries and regions. Ultimately, the validation of the T-LOC scale in Lebanon is pivotal for advancing both local and international road safety initiatives, tailoring interventions to the specific psychological dynamics influencing driving behavior in the Lebanese context.

### Aims of the study

This study aimed to adapt the Traffic Locus of Control (T-LOC) scale to Lebanese culture and assess its psychometric properties to ensure its applicability in the Lebanese context. Additionally, it sought to explore the association between T-LOC and driving behavior, as well as its correlation with involvement in traffic crashes or receiving traffic offenses.

## II. Methods

The validation of the Arabic version of T-LOC (T-LOC-A) was conducted through a two-stage process. The first stage involved translating and culturally adapting the T-LOC, and the second stage focused on testing its psychometric properties (**Table 1**).

**Table 1:** Proccesses for cross-cultural adaptation and validation of t-loc among lebanese drivers.

Process	Steps	Description
**Translation and cross-cultural adaptation**	Translation	Translation of the T-LOC questionnaire into Lebanese Arabic by two independent bilingual translators.
	Synthesis	Collaboration between translators to reconcile discrepancies and produce a synthesized version of the translation.
	Back-Translation	Translation of the synthesized version back into English by two independent translators
	Review by Committee	Assessment of the translated version’s clarity and appropriateness for the Lebanese context by a committee comprising a road safety specialist, linguistic professional, psychologist, principal investigator, and three drivers from different age groups.
	Pilot Testing	Evaluation of the pre-final version (T-LOC) on a small convenience sample of 35 drivers from different age groups and Lebanese governorates
**Reliability**	Internal Consistency Reliability	Assessment of internal consistency using Cronbach’s alpha.
	Test-Retest Reliability	A subset of 40 individuals was asked to complete the questionnaire once again after an interval of approximately 3 weeks
**Validity**	Content Validity	Using qualitative and quantitative approaches, including the Lawshe method and calculation of Content Validity Index (CVI).
** **	Construct Validity	Kaiser-Meyer-Olkin (KMO) and Bartlett tests, factor analysis, and Confirmatory Factor Analysis (CFA) with goodness-of-fit indices.

### Translation and cross-cultural adaptation process

Permission was secured from the corresponding author to translate the original T-LOC questionnaire into Arabic as part of a comprehensive project, which also involves the validation of other scales pertinent to driver behavior in the Lebanese context. Following the steps outlined by Beaton et al. [20], the translation and cross-cultural adaptation of the T-LOC-A scale were meticulously conducted. The 16-item T-LOC underwent a rigorous translation process into Lebanese Arabic, engaging two independent bilingual translators and utilizing a synthesis approach [20]. It’s noteworthy that the methodology closely paralleled the one we employed for the validation of the driver behavior questionnaire (DBQ) [21].

One translator, specializing in road safety, and another from the language department collaborated to translate and reconcile discrepancies by consensus, producing a synthesized version. Subsequently, this translation underwent back-translation into English by two independent translators lacking behavioral science expertise.

A committee, comprising a road safety specialist, linguistic professional, psychologist, and the principal investigator, and three drivers from different age groups reviewed and assessed the translated version’s clarity and appropriateness for the Lebanese context. Following committee feedback, all items were retained. The pre-final version, T-LOC, was piloted on a small convenience sample of 35 drivers from different age groups and from all Lebanese governorates and to evaluate comprehensibility, resulting in minor revisions based on participant feedback. These adjustments ensured the ultimate Arabic version of T-LOC was linguistically suitable for Lebanese drivers, marking its readiness for subsequent psychometric testing. Of note, the pilot study’s sample size of 35 drivers was chosen considering feasibility and practical constraints, like limited resources and time. Typically, a sample size of 30–50 participants suffices to identify major issues with the instrument being tested. Hence, this size balances feasibility and meaningful insights into the translated instrument’s quality. While smaller than the main study, it adequately serves its purpose of identifying potential issues and refining the instrument before full validation.

### Psychometric testing

#### Study design and participants

In the timeframe spanning October to December 2019, a cross-sectional study was conducted targeting the diverse landscape of Lebanese drivers across all governorates. Using a convenience sampling approach, participants were meticulously chosen based on both accessibility and eagerness to partake in the study. Subsequent data underwent meticulous weighting, factoring in critical demographic aspects such as gender, age, and dwelling region. This intricate process ensured a nuanced representation mirroring the Lebanese population’s age, location, and gender distribution. Target figures were precisely outlined for each governorate—Bekaa, Baalbeck-Hermel, Mount-Lebanon, Beirut, North, Akkar, South, and Nabatyeh—drawing from figures provided by the Central Administration of Statistics. With an official list of drivers proving elusive, potential respondents were sought in bustling public spaces like shopping areas and parking stations. Eligibility criteria included active Lebanese drivers aged 18 or above, holding a valid driver’s license, engaging in regular driving activities, and expressing a willingness to participate. Exclusions were made for those not currently involved in driving, illiterate individuals unable to comprehend the questions, and non-Lebanese drivers.

#### Sample size calculation

Guidance on sample size suggests that having 300 or 5–10 participants for each scale item is sufficient to establish evidence of scale validity and reliability [22]. Given that the T-LOC comprises 16 items, the minimum required participants would be in the range of 80–160. To enhance study power and minimize sampling error, a preliminary estimate was made by multiplying the calculated sample size by 3.5, resulting in a final sample size of 560 participants. It’s important to note that the individual responsible for data entry had no involvement in the data collection process.

#### Ethical considerations

Written informed consent was obtained for each participant. They were reassured that their participation was voluntary and that they were free to withdraw at any time. The study was designed to prioritize the protection of participants. All information was collected and handled with strict confidentiality measures in place to ensure anonymity. This study was conducted following the ethical principles outlined in the Declaration of Helsinki. The protocol of the study was reviewed by the higher technical school. It was exempted from ethical approval as it is a low-risk health study and caused no plausible harm or stigma to participants. Importantly, the study did not involve any clinical data about patients nor was it designed as a clinical trial.

#### Data collection tool

A standardized anonymous questionnaire was developed in Arabic, the native language of Lebanon using closed-ended questions. The average completion time was approximately 10 minutes. The introductory page provided a brief overview of the survey’s background and objectives, along with instructions for filling out the questionnaire. The questionnaire consisted of three sections:

1. The first section included questions assessing socio-demographic characteristics of the study participants, such as age, gender, education level, marital status, and working status.
2. The second section included exposure variables such as driving experience and annual mileage and risk involvement (being involved in RTCs or receiving traffic offenses during the previous three years).
3. The third section included two scales:

**The T-LOC questionnaire** comprises 16 items, with 5 items specifically addressing self-related factors, such as personal risk-taking behaviors, 6 items targeting other drivers’ behavior, such as their level of risk-taking, 3 items target vehicle/environment (e.g. mechanical failure in the car), and 2 items target fate (e.g. bad luck). For each item, participants were asked to rank the possibility that each of these 16 reasons could be the cause of RTCs on a five-point scale (1 = not at all possible; 5 = highly possible) [8].

**The Driver behavior questionnaire (DBQ)** is one of the most widely used instruments in traffic psychology developed by Reason [23]. The DBQ assessed aberrant driver behavior by asking how often they experience specific types of aberrant driving behaviors on a six-point scale (1 = never; 2 = hardly ever; 3 = occasionally; 4 = quite often; 5 = frequently; 6 = nearly all the time) across different driver situations. The DBQ questionnaire has four components; ordinary violations, aggressive violations, errors, and lapses. The DBQ questionnaire was previously validated among Lebanese drivers by Youssef et al. [21], with a focus on assessing driver aberrant behavior on the road. In this study, the Arabic version of the DBQ was utilized. Strong internal reliabilities for both the aggressive violations subscale (0.89) and the ordinary violations subscale (0.85) were found.

#### Data collection procedure

After receiving the signed informed consent, eligible respondents were asked to complete a questionnaire via a face-to-face approach. The distribution of questionnaires was overseen by two proficient data collectors in each governorate, both students specializing in traffic studies at the Lebanese Higher Technical School. Prior to involvement, the data collectors orally communicated the study’s objectives and provided general instructions to the participants. It’s noteworthy that drivers were under no obligation to participate and received no financial incentives for their involvement. To assess test-retest reliability, a subset of 40 individuals was asked to complete the questionnaire once again after an interval of approximately 3 weeks [24]. To ensure the reliability of the test-retest process, a minimum sample size calculation was conducted using a specific formula: n = $\frac{2\times Z2\times SD2}{(\Delta /\mu )2}$, where n = required sample size, Z = Z-score corresponding to the desired level of statistical power, SD = standard deviation of the measurement, Δ = desired level of precision or margin of error. The formula yielded a minimum required sample size of 36 participants.

### Statistical analysis

The data collected for this study was entered into the Statistical Package for Social Sciences (SPSS), version 24.0, and analyzed using its built-in statistical tools. To ensure the accuracy of data entry, the person responsible for entering the data into SPSS was not involved in the data collection process. Given that missing values constituted < 10% of the total data, they were not substituted.

In terms of reliability, the study assessed the internal consistency reliability of the T-LOC using Cronbach’s alpha, considering a value of α ≥ 0.70 as satisfactory [25, 26]. Test-retest reliability was also examined, involving 40 drivers completing the questionnaire twice after almost 3 weeks. The test-retest reliability of the scale was determined using the intra-class correlation coefficient (ICC). ICC values between 0.40 and 0.59 are considered fair, values between 0.60 and 0.74 good, and values between 0.75 and 1.0 excellent [27].

In terms of validity, content validity was determined through both qualitative and quantitative approaches, involving a panel of eight experts. The quantitative method utilized the Lawshe method [28] to calculate the content validity ratio (CVR), with a CVR of 0.49 or higher considered acceptable [28]. The Content Validity Index (CVI) was calculated as the mean score of retained items with a CVR of 0.49 or higher [29]. Regarding construct validity, the Kaiser-Meyer-Olkin (KMO) and Bartlett tests were conducted before initiating factor analysis. The original dataset was divided into two roughly equal samples, with the first sample employed for Principal Component Analysis and factor analysis with Varimax rotation to assess the validity of the 16-item T-LOC for the Lebanese population and identify T-LOC factors The determination of factors included in the model was based on Eigenvalues >1 and the scree plot. Additionally, Then, a parallel bootstrapping analysis (PA) was performed to derive simulated eigenvalues from random samples for comparing with the observed data and to determine the number of components or factors to retain from factor analysis. Confirmatory Factor Analyses (CFA) were executed using IBM AMOS 24.0, with reported fit indices and corresponding cutoffs for goodness of fit. The structural models were deemed satisfactory when the Chi-squared value (χ 2) /degree of freedom (χ2/df < 5), Comparative Fit Index (CFI > 0.9), Tucker Lewis Index (TLI) (≥ 0.95), Root Mean Square Error of Approximation (RMSEA < 0.08), and Standardized Root Mean Square Residual (SRMR < 0.08) met specified criteria [30]. In cases of poor fit, modification indices were explored to enhance model fit. The modified model allowed for freely estimated covariances, and cross-loading items (those loading 0.40 on two or more factors) were excluded.

Descriptive analyses were performed using frequency and percentage for categorical variables and mean and standard deviation for continuous variables. A bivariate analysis was conducted using the Chi-square test to check for associations between categorical variables and the ANOVA test to compare the means of LOC subscales and the categorical variables. Linear correlation analysis between continuous variables was performed using the Pearson correlation coefficient. All variables that showed a *p* < 0.2 in the bivariate analysis were included in the model as independent variables. Finally, logistic regression analyses were performed to explore the association between T-LOC scores and risk involvement and driver behavior.

## III. Results

### Translation and content validity

Following a thorough examination of the T-LOC-A translation and back-translation, and a pilot test involving 35 drivers, minor adjustments, including the clarification of some ambiguous terms, were incorporated to finalize the Arabic version. The back-translated variant exhibited a high degree of similarity to the original document. Two experts were consulted, and their unanimous agreement affirmed the instrument’s suitability for gauging the driver’s locus of control (LOC). They appraised the questionnaire, attesting to its commendable content and face validity. Every item garnered a Content Validity Ratio (CVR) surpassing 0.75, validating the appropriateness of all questionnaire items, which were consequently retained. Moreover, the overall Content Validation Index (CVI) score for T-LOC stood at 0.88, indicative of robust content validity for the scale.

### Socio-demographic characteristics of participants

Of the 568 drivers who participated (Table 2), the majority were male (69%) and aged between 30- and 49-years old (42.1%). More than half of the participants (52.6%) were married and 62.1% were living in urban areas; 58% of the drivers held a university degree or above. Out of the total, 41.0% of surveyed drivers were involved in RTCs in the previous three years and half of them (50.4%) had received at least one ticket in the previous three years.

**Table 2:** Socio-demographics characteristics of the study sample (N = 568).

	n	%
**Gender **		
Male	392	69.0
Female	176	31.0
**Age groups (years)**		
Less than 29	250	44
30–49	239	42.1
50 and above	79	13.9
**Marital status **		
Single	239	42.1
Married	299	52.6
Other (Widowed, Separated. . .)	30	5.2
**Education level **		
Secondary or less	238	41.9
University or above	330	58.1
**Occupation**		
Non-professional driver	504	88.7
Professional driver (taxi)	64	11.3
**Annual mileage **		
<6 000Km	278	48.9
≥6000 Km	290	51.1
**Road traffic crashes in the previous 3 years (mean ± SD)**	0.87±1.47	
**Fines last three years (mean ± SD)**	0.46±0.498	

N: Frequency, %: Percentage, SD: Standard deviation

### Factor structure of the T-LOC scale

#### Exploratory factor analysis

The exploratory factor analysis conducted on the T-LOC scale demonstrated a Kaiser-Meyer-Olkin (KMO) measure of 0.837, indicating sufficient sampling adequacy In addition, Bartlett’s Test of Sphericity was highly significant (p <0.001), further indicating that the factor analysis was appropriate. The scree plot of the Eigenvalues revealed a four-factor structure of the T-LOC scale. The scree plot of the Eigenvalues indicated that the T-LOC scale had a four-factor structure, which accounted for 78% of the total variance. The eigenvalues derived from the bootstrapping procedure confirmed the selection of four factors with eigenvalues over 1 (eigenvalues of 6.244 and 1.407). The first factor, which explained 39.0% of the total variance, consisted of six items measuring causes attributed to other drivers (items 3, 4, 8, 10, 14, 15 as presented in Table 3). Therefore, this factor was named "other drivers". The second factor, accounting for 15.7% of the variance, comprised five items that measured drivers’ self-based causes, such as personal skills and behavior. This factor was named "self".

**Table 3:** Exploratory factor analysis of the T-LOC scale among Lebanese drivers.

	T-LOC scale items	T-LOC components			
	
Item		Other Drivers	Self	Vehicle & Environment	Fate
LOC15	Other drivers’ dangerous overtaking	0.957			
LOC14	Other drivers driving under influence of alcohol	0.945			
LOC8	Other drivers drive often with too high speed	0.944			
LOC4	Other drivers’ risk-taking while driving	0.912			
LOC10	Other drivers drive too close to my car	0.902			
LOC3	Shortcomings in other drivers’ driving skills	0.842			
LOC1	Shortcomings in my driving skills		0.887		
LOC16	My own dangerous overtaking		0.886		
LOC2	My risk-taking while driving		0.809		
LOC9	If I drive too close to the car in front		0.770		
LOC7	If I drive often with too high a speed		0.593		
LOC12	Bad weather or lighting conditions			0.897	
LOC6	Dangerous roads			0.885	
LOC13	Mechanical failure in the car			0.870	
LOC11	Fate				0.836
LOC5	Bad luck				0.831
E	Eigenvalue	6.244	2.515	2.316	1.407
Α	Chronbach alpha	0.907	0.883	0.859	0.657
V	Variance	39.024	15.718	14.473	8.797

The third factor accounted for 14.5% of the variance and included three items measuring causes attributed to the vehicle and the environment. This factor was named "vehicle and environment". The fourth factor, which accounted for 8.8% of the variance, consisted of two items measuring causes attributed to fate and chance. This factor was named "fate".

#### Confirmatory factor analysis

A Confirmatory Factor Analysis (CFA) was conducted to establish the multidimensional model of the T-LOC scale. The hypothesized model, consisting of 20 items, was initially proposed to load into four factors based on the Exploratory Factor Analysis (EFA). However, the model displayed inadequate fit indices (χ2/df = 7.226; NFI, CFI, and GFI<0.9, SRMR = 0.05, RMSEA = 0.146).

**Fig 1:**
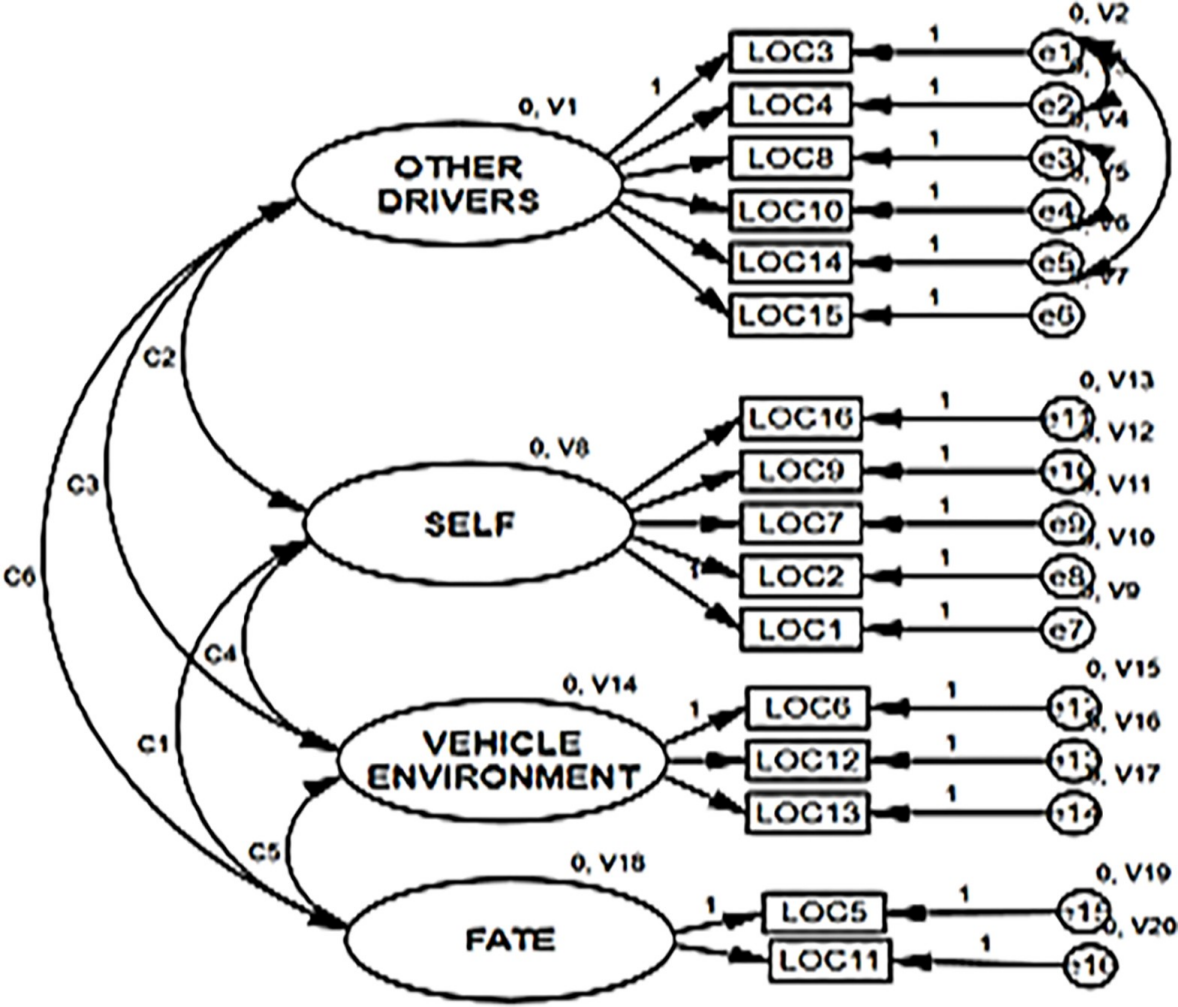
Multidimensional factor structure of the arabic version of the Traffic Locus of Control (T-Loc) scale.

Further inspection of the modification indices indicated that adding error covariance between e2 and e1, e3 and e4, and e1 and e5 could improve the model fit. After implementing these modifications, the fit indices significantly improved (χ2/df = 2.302 <5; NFI = 0.953, CFI = 0.973, GFI = 0.917, AGFI = 0.902, RMSEA = 0.038 and SRMR = 0.042<0.05), confirming the adequacy of the model (Fig 1).

#### Reliability of the T-LOC-A scale

The overall reliability of the T-LOC-A scale was good (α = 0.85). Alpha reliabilities for these subscales ranged from 0.66 to 0.91. Skewness (-0.82 to 0.0.4] and kurtosis (-0.28 to 0.67] estimates for the four factors allowed the use of parametrical correlational analyses (Table 4).

**Table 4:** Mean scores, Cronbach’s alpha coefficient, skewness, and kurtosis of the T-LOC-A scale.

		All drivers (N = 568)						
		Scale Mean	S.D	Min	Max	Alpha α	Skewness	Kurtosis
**Self-related**		17.08	3.79	7	25	0.88	-0.50	0.28
**Other drivers**		22.74	5.31	7	30	0.91	-0.82	0.61
**Fate**		5.87	1.98	2	10	0.66	-0.40	-0.55
**Vehicle/ Env.**		10.29	2.04	3	15	0.86	-0.69	1.67
** T-LOC-A scale**		56.21	8.42	28	71	0.85	-0.57	0.47

#### Correlation between the traffic locus of control dimensions and the DBQ subscales

The correlations between T-LOC-A factors ranged from 0.11 to 0.40, most of them being statistically significant (p<0.05) with a low to very low correlation (Table 5). The results indicated that the other drivers’ dimension of T-LOC-A was positively correlated with aggressive violations (r = 0.387, p<0.05). Notably, the self-dimension, representing internal T-LOC-A, was also positively correlated to aggressive violations (r = 0.155, p<0.05). Furthermore, the fate dimension showed positive correlations with aggressive violation (r = 0.11, p<0.001) and lapses (r = 0.217, p<0.05). Additionally, the other drivers’ subscale was positively correlated with errors (r = 0.320, p<0.05), while the vehicle and environment subscale was found to be correlated with lapses (r = 0.131, p<0.05).

**Table 5:** Correlation between the traffic locus of control dimensions and the DBQ subscales.

	Self-related	Other drivers	Vehicle and environment	Fate
**Self-related**	1	-.401**	0.13*	-0.11*
**Other drivers**	-.401**	1	0.01	-0.06
**Vehicle and environment**	0.13*	0.01	1	-0.02
**Fate**	-0.11*	0.06	-0.02	1
**Aggressive violation**	0.155*	.387*	0.057	.110**
**Ordinary violation**	-0.019	-0.012	-0.09	0.047
**Errors**	0.037	0.320*	0.038	0.006
**Lapses**	0.009	-0.026	0.031*	0.217*

Note

* Correlation is significant p<0.05

** Correlation is significant at the 0.001 level (p<0.001)

#### Association between T-LOC-A and socio-demographic variables

Table 6 displays the gender differences in T-LOC-A subscales. Men tended to attribute responsibility for driving situations to other drivers more than women (Mean 22.69 (SD = 4.95) for men versus Mean 23.45 (SD = 5.19) for women, p = 0.024, Cohen’s d = 0.21). On the other hand, women believed more in fate and luck than men did (Mean 5.84 (SD = 2.06) for men versus Mean 6.24 (SD = 1.81) for women, p = 0.012, Cohen’s d = 0.30). However, no significant differences were observed between men and women in the "self" and "vehicle and environment" subscales. Moreover, fate was negatively correlated with age (r = -0.149, p<0.001) and years of experience (r = -0.198, p<0.001) and was also associated with annual mileage (p<0.001). The "other drivers" scale was positively correlated with all variables in the analysis, including age (r = 0.098, p<0.05), years of experience (r = 0.128, p<0.001), and annual mileage (p = 0.007). Finally, the vehicle/environment subscale was positively related to annual mileage (p = 0.029).

**Table 6:** Arabic traffic locus of control by sociodemographic characteristics.

	Gender		P-value
	Male (N = 392)	Female (N = 176)	
	N(SD)	N(SD)	
**Self**	17.25(3.74)	16.7(3.90)	0.26
**Other drivers**	22.69(4.95)	23.45(5.19)	0.024
**Vehicle & environment**	10.3(2.16)	10.28(1.76)	0.13
**Fate**	5.84(2.06)	6.24(1.81)	0.012
	**Annual mileage**		
** **	**≤6000 km (N = 278)**	**>6000 Km (N = 290)**	
**Self**	17.02(3.67)	16.93(3.78)	0.586
**Other drivers**	21.49(4.33)	24.63(5.12)	0.007
**Vehicle & environment**	9.01(2.11)	11.18(3.05)	0.029
**Fate**	5.09(3.03)	7.21(2.71)	<0.001
	**Age**	**Pearson r**	**P-value**
**Self**	** **	0.051	p>0.05
**Other drivers**	** **	0.098	P<0.05
**Vehicle & environment**	** **	-0.103	p>0.05
**Fate**	** **	-0.198**	p<0.001
	**Year of experience**		
**Self**	** **	0.028	p>0.05
**Other drivers**	** **	0.128**	p<0.01
**Vehicle & environment**	** **	0.151**	p<0.01
**Fate**	** **	-0.125**	p<0.01

N.B

**: p<0.01

*: p<0.05

#### Traffic locus of control and road traffic crashes

The logistic regression analysis revealed significant associations between various factors related to Traffic Locus of Control (T-LOC) and the likelihood of driver involvement in Road Traffic Crashes (RTCs) (Table 7). Notably, higher annual mileage (>6000 Km) was positively associated (p-value = 0.048, aOR = 2.147), with the risk of being involved in RTCs. Drivers covering distances exceeding 6000 km were more prone to RTCs compared to those covering 0–6000 km. Furthermore, the "LOC self" factor exhibited a significant protective effect (p<0.001, aOR = 0.922), suggesting that as the "LOC self" score increases, the odds of RTC involvement decrease. Conversely, the "LOC other drivers" factor showed a highly significant association (p-value < 0.001) with increased odds of RTC involvement (aOR = 1.205). Additionally, the "LOC vehicle/environment" factor demonstrated a significant association (p-value = 0.019), with higher scores associated with increased odds of RTC involvement (aOR = 1.109).

**Table 7:** Multivariable logistic regression of the T-LOC factors associated with the drivers involvement in RTCs.

Involvement in RTC						
	No	Yes	P-value	aOR	95% C.I. for aOR	
	N (%)	N (%)			Lower	Upper
**Gender**			0.732			
Male	233(59.4%)	159(40.6%)				
Female	104(59.1%)	72(40.9%)				
**Occupation**			0.074			
Student	85(62%)	52(38%)				
Driver	28(43.8%)	36(56.3%)				
Working but not as driver	196(61.3%)	124(38.8%)				
Not working	28(59.6%)	19(40.4%)				
**Age groups (years)**			0.097			
Less than 20	28(63.6%)	16(36.4%)				
20–29	127(61.7%)	79(38.3%)				
30–39	85(62%)	52(38%)				
40–49	56(54.9%)	46(45.1%)				
50 and above	41(51.9%)	38(48.1%)				
**Educational level**			0.745			
12 years or less	137(57.6%)	101(42.4%)				
>12 years	200(60.6%)	130(39.4%)				
**Annual Mileage (Km)**			0.048	2.147	1.110	4.153
0–6000 km	181(65.1%)	97(34.9%)				
>6000 km	156(53.8%)	134(46.2%)				
**Year of driving experience**			0.123			
10 years or less	205(61.9%)	126(38.1%)				
More than 10 years	132(55.7%)	105(44.3%)				
	**Mean**	**SD**				
**LOC fate**	5.87	1.98	0.568			
**LOC self**	17.080	3.790	0.001	0.922	0.877	0.970
**LOC other drivers**	22.740	5.310	<0.001	1.205	1.062	1.849
**LOC vehicle/environment**	10.29	2.04	0.019	1.109	1.017	1.209

N.B: Educational level of 12 years is equivalent to secondary level, *p-value<0.05 is considered significant

#### Traffic locus of control and traffic offenses

Age groups, occupation and educational levels did not show significant correlations with traffic offenses, implying uniform likelihoods across different age and educational categories. In contrast, annual mileage emerged as a highly significant factor, revealing that drivers covering more than 6000 km had significantly higher odds of committing offenses compared to their counterparts covering 0–6000 km. Additionally, the number of years of driving experience did not exhibit a significant association with traffic offenses. Regarding the Locus of Control (LOC) factors, "LOC fate" displayed a significant association with traffic offenses, suggesting that higher scores increased the likelihood of offenses. Conversely, "LOC self" exhibited a protective effect, with higher scores correlating with decreased odds of committing offenses. However, "LOC other drivers" and "LOC vehicle/environment" did not show significant associations with traffic offenses (Table 8).

**Table 8:** Multivariable logistic regression of the T-LOC factors associated with getting traffic offences.

	Traffic offenses in the past 3 years			
	P-value	aOR	95% C.I. for aOR	
			Lower	Upper
**Gender**	0.332			
Male				
Female				
**Occupation**	0.074			
Professional driver				
Non-professional driver				
**Age groups (years)**	0.736			
Less than 29				
30–49				
50 and above				
**Educational level**	0.449			
12 years or less				
>12 years				
**Annual Mileage (Km)**	<0.001	4.071	2.212	7.493
0–6000 km				
>6000 km				
**Year of driving experience**	0.189			
10 years or less				
More than 10 years				
**LOC fate**	0.015	1.117	1.022	1.220
**LOC self**	0.019	0.949	0.908	0.991
**LOC other drivers**	0.593			
**LOC vehicle/environment**	0.807			

N.B: Educational level of 12 years is equivalent to secondary level, *p-value<0.05 is considered significant

## IV. Discussion

### Main findings

This research was designed to adapt and validate an effective tool for measuring T-LOC among Lebanese drivers. Our findings indicate that the T-LOC-A has adequate psychometric properties revealing good to excellent reliabilities. The results from the exploratory factor analysis showed that the factorial structure of the T-LOC-A was similar to that of the original version [8] revealing the same multidimensional structure with four subscales namely internal locus of control called “self”, “others drivers”, “fate”, and “vehicle/environment”. All four factors had acceptable reliability. Its multidimensional structure is statistically supported by satisfactory fit indices. The factorial solution of T-LOC-A is consistent with the four broad facets found in the Chinese [31], Swedish [9], and Romanian [10] versions, despite variations in factorial structure and content revealed by the number of items [10]. Furthermore, the differentiation between external and internal beliefs is consistent with previous studies [4, 7, 8, 32–34]. Our study demonstrated that the correlations between the internal and external T-LOC factors were low to moderate, which is in line with previous studies on T-LOC scale development [7, 8, 10].

The pattern of correlations between the T-LOC-A factors was mostly similar to those reported in previous studies [9, 16]. Specifically, we observed that other drivers and fate were negatively correlated with self [10], while self was positively correlated with the vehicle/environment dimension. These results are consistent with earlier research that also found a positive correlation between the self-scale and the vehicle/environment dimension [8–10]. However, our study did not find any significant correlation between the external dimensions, which is in contrast to previous research indicating that other driver’s dimensions were positively correlated with fate and vehicle/environment, and that fate was positively correlated with vehicle/environment [10]. Further research is needed to explain these particular findings among Lebanese drivers.

Our findings revealed that there were significant gender differences in how responsibility for RTCs was attributed to internal or external factors. Specifically, men reported a higher tendency to attribute the responsibility for different driving situations and RTCs to the other drivers, while women believed more in fate and bad luck as a contributor to RTCs. Our results are similar to the findings of a study conducted among Romanian drivers, that revealed the same pattern of responsibility attributions between men and women [7]. Considering self and vehicle-environment scales, the results did not reveal significant differences between men and women. These results suggest that gender may play a role in the attributional processes related to driving situations and RTCs and that interventions designed to improve road safety should take into account gender differences in attributions of responsibility.

Of note, the present study revealed that Lebanese female drivers had the highest scores in all the T-LOC-A factors compared to male drivers. These results were also in line with previous studies [5, 8] and in some cases, similar to the findings in other fields, such as health psychology [35].

Remarkably, this study has revealed a positive correlation between the concept of "self" and aggressive driving behavior. This suggests that Lebanese drivers who tend to take personal responsibility for traffic accidents are more likely to engage in aggressive violations while driving. These findings align with previous studies that have shown a link between an internal locus of control and risky driving behaviors. Such behaviors may stem from drivers’ overly optimistic beliefs about their ability to avoid accidents [4, 5].

In addition, our study revealed that drivers who tended to attribute the causes of RTCs to “other drivers” were also more likely to engage in dangerous driving behavior, revealed by the aggressive violation. This might be explained by the fact that drivers who are anxious and about other drivers’ performances and actions on the road are more likely to react impulsively and adopt risky behaviors revealed by aggressive violations. Furthermore, we found that the belief in fate or luck as a cause of RTCs was also associated with aggressive driving behavior. These drivers tended to absolve themselves of responsibility for accidents and were more likely to engage in dangerous driving behavior. Notably, we observed a weak or non-existent correlation between T-LOC subscales and lapses, which explains why lapses were not included in the analysis of the relationship between aberrant behavior and T-LOC. Interestingly, the fate dimension was also correlated to the aggressive violation. Drivers who blamed destiny or luck to be the causes of RTCs, tended to absolve themselves of responsibility for accidents and were more likely to engage in dangerous driving behavior. Notably, the weak or non-existent correlation T-LOC subscales and lapses explains why lapses were not included in the analysis of the relationship between aberrant behavior and T-LOC.

Concerning the association between the T-LOC-A factors with accident involvement and traffic offences, the results highlighted a positive association between the dimensions of external T-LOC scale (other drivers, vehicle-environment) and being involved in RTCs in the last three years. The "LOC self" factor emerged as a significant protective factor, indicating that as drivers’ internal control perception increases, the likelihood of RTC involvement decreases. Our results are consistent with the findings of Özkan & Lajunen, that found that self can significantly predict drivers’ total number of offenses and that involvement in active accidents was associated with internality [36, 37]. However, the non-significant correlation between self and traffic crashes was found other studies [10]. Given the inherent link between being responsible for vehicle accidents and experiencing personal culpability, it is conceivable that our participants might not have veraciously disclosed their engagement in road traffic crashes. This potential inconsistency could be attributed to a conscious effort to evade the emotional repercussions of personal guilt, social stigma, or other adverse emotional states associated with recollecting such incidents.

In contrast, the "other drivers" and the "vehicle/environment” factors exhibited a robust association with an increased likelihood of road traffic crash (RTC) involvement, highlighting the substantial impact of external factors on crash risk. These findings underscore the intricate nature of road safety, emphasizing the imperative consideration of both internal and external locus of control factors in devising targeted interventions and strategies to mitigate RTC risks. This phenomenon could be elucidated by evidence from various studies that suggests a correlation between attributing causes of RTCs to external factors and a diminished inclination to adopt precautionary measures [7]. However, it is apparent that respondents who have experienced RTCs and subsequent traffic offenses may consciously deflect responsibility. Consequently, they may be inclined to attribute the causes of road traffic accidents to external factors when responding to the T-LOC-A scale. Our findings align with the research of Holland et al., 2010 [4, 5] and Özkan & Lajunen, 2005 [36, 37].

Turning to the Locus of Control (LOC) factors and traffic offenses, "LOC fate" displayed a significant association with traffic offenses, suggesting that drivers attributing events to external forces are more likely to engage in offenses. Drivers who find more external causes of accidents seem to have more traffic offences, which might be explained by the fact that these individuals are less careful and take less actions to prevent traffic accidents [7]. However, drivers who were involved in traffic offences may avoid presenting themselves as responsible for RTCs involvement, which may have led them to attribute road RTCs to external causes when responding to the T-LOC [4, 5]. In contrast, the "LOC self" factor exhibited a protective effect, indicating that as drivers’ internal control perception increases, the odds of committing offenses decrease. However, "LOC other drivers" and "LOC vehicle/environment" did not show significant associations with traffic offenses, suggesting that the impact of these external factors on traffic violations might be less pronounced in this context. These nuanced findings highlight the need for a comprehensive understanding of the interplay between individual characteristics, driving behaviors, and external influences in the context of traffic offenses.

### Strengths

This is the first national study conducted in Lebanon to assess traffic locus of control among drivers. Its main objective is to test the T-LOC scale in a sample that is different from the one used in its initial development. Therefore, the sample of Lebanese drivers in this study is considered to be sufficient as participants come from various geographic areas and drive in different traffic environments compared to the Turkish drivers in the original study [8]. Moreover, this study allows a close approximation of the findings to the general driver population, particularly since no prior studies have taken into account a representative sample from all regions in Lebanon. By conducting this study, we provide an opportunity for traffic safety researchers in Arabic countries to perform further studies in various work settings and ensure an objective assessment of traffic locus of control.

### Limitations

Several limitations should be acknowledged in interpreting the findings of this study. Firstly, the cross-sectional design employed restricts our ability to establish causal relationships between the identified associations. While the study aimed to control for various factors, including gender, age, and geographical regions, the use of convenience sampling might introduce selection bias. Additionally, relying on self-report measures for data collection, although common in similar studies, introduces the potential for recall and information biases. The absence of significant relationships between desirability and study variables indicates that the observed results are not likely influenced by social desirability. However, it is essential to recognize the inherent limitations of self-reported responses. Moreover, the study focused on the locus of control without incorporating measures of driving skills, confidence, and optimism bias, which could provide a more comprehensive understanding of the factors influencing traffic behavior [8],. Finally, while the study sheds light on the traffic locus of control among Lebanese drivers, generalizability to other cultural contexts should be approached with caution, emphasizing the need for further cross-cultural validation.

### Implications

This study carries profound implications for the field of traffic psychology, significantly advancing our understanding of traffic locus of control. The introduction of the Arabic version of the T-LOC (T-LOC-A) as a valid and reliable instrument holds practical value for practitioners aiming to reduce accident risk and enhance road safety in Lebanon. The study’s unique contribution lies in its provision of a comprehensive platform for traffic safety researchers in Arabic countries, encouraging further investigations in diverse work settings. The findings underscore the need for targeted interventions, particularly for novice drivers, emphasizing the positive impact of developing an internal locus of control on reducing risky driving behavior. The study’s exploration of the interplay between demographic variables and behavior in the road traffic domain contributes to a more nuanced understanding, paving the way for holistic interventions. By elucidating the relationship between T-LOC and road traffic crashes, the study not only enriches academic knowledge but also provides actionable insights for mitigating accident risk in Lebanon and potentially in other Arabic countries. Furthermore, the cultural adaptation of the T-LOC scale emphasizes the importance of considering cultural specificities in psychological assessments, ensuring the tool’s relevance and effectiveness in the Lebanese context.

## V. Conclusion

The Arabic version of T-LOC (T-LOC-A) is a valid and reliable instrument in the Lebanese driving context and cultural specificities. The factorial structure of T-LOC-A aligns with that of the original T-LOC, indicating that the tool is reliable in measuring driving locus of control. The use of T-LOC-A can aid in the development of interventions aimed at reducing the likelihood of road traffic crashes among Lebanese drivers who exhibit risky driving behavior. Future studies examining the relationship between T-LOC and involvement in road traffic crashes are strongly recommended.

## Supporting information

S1 Questionnaire (DOCX)

## Abbreviations

T-LOC-A: Traffic locus of control scale–Arabic version
RTCs: Road traffic crashes
T-LOC: The Traffic Locus of Control scale
P: P-value
SD: Standard deviation
SPSS: Statistical Package for Social Sciences
CFA: Confirmatory factor analysis
EFA: Exploratory factor analysis
KMO: Kaiser–Meyer–Olkin
CVI: Content Validity Index
CVR: Content validity ratio
RMSEA: Root mean square error of approximation
SRMR: Standardized root mean square residual
GFI: Goodness-of-Fit Index
CFI: Comparative Fit Index
NFI: Normed Fit Index
DBQ: Driver behavior questionnaire
α: Cronbach’s alpha
r: Pearson Correlation Coefficient
d: Cohen’ d
IPNET: Lebanese Higher Institute of Technical & Professional

## References

[1] Rumar K., Driver requirements and road traffic informatics. Transportation, 1990. 17(3): p. 215–229.

[2] Rotter J.B., Generalized expectancies for internal versus external control of reinforcement. Psychological Monographs: General and Applied, 1966. 80(1): p. 1–28. 5340840

[3] Rotter J.B., Some problems and misconceptions related to the construct of internal versus external control of reinforcement. Journal of Consulting and Clinical Psychology, 1975. 43(1): p. 56–67.

[4] Arthur W. and Doverspike D., Locus of control and auditory selective attention as predictors of driving accident involvement: A comparative longitudinal investigation. Journal of Safety Research, 1992. 23(2): p. 73–80.

[5] Holland C., Geraghty J., and Shah K., Differential moderating effect of locus of control on effect of driving experience in young male and female drivers. Personality and Individual Differences, 2010. 48: p. 821–826.

[6] Özmen O. and Sümer Z.H., Predictors of risk-taking behaviors among Turkish adolescents. Personality and Individual Differences, 2011. 50: p. 4–9.

[7] Montag I. and Comrey A.L., Internality and externality as correlates of involvement in fatal driving accidents. Journal of Applied Psychology, 1987. 72(3): p. 339–343. 3624153

[8] Özkan T. and Lajunen T., Multidimensional Traffic Locus of Control Scale (T-LOC): Factor structure and relationship to risky driving. Personality and Individual Differences, 2005. 38: p. 533–545.

[9] Warner H.W., Ozkan T., and Lajunen T., Can the traffic locus of control (T-LOC) scale be successfully used to predict Swedish drivers’ speeding behaviour? Accid Anal Prev, 2010. 42(4): p. 1113–7. doi: 10.1016/j.aap.2009.12.025 20441820

[10] Mairean C., et al., Traffic locus of control scale–Romanian version: Psychometric properties and relations to the driver’s personality, risk perception, and driving behavior. Transportation Research Part F Traffic Psychology and Behaviour, 2017. 45: p. 131–146.

[11] Phares E.J. and Wilson K.G., Responsibility attribution: role of outcome severity, situational ambiguity, and internal-external control. J Pers, 1972. 40(3): p. 392–406. doi: 10.1111/j.1467-6494.1972.tb00069.x 5069795

[12] Peterson C. and Stunkard A.J., Cognates of personal control: Locus of control, self-efficacy, and explanatory style. Applied and Preventive Psychology, 1992. 1(2): p. 111–117.

[13] Strickland B.R., Delay of gratification and internal locus of control in children. J Consult Clin Psychol, 1973. 40(2): p. 338. doi: 10.1037/h0034499 4694214

[14] Hoyt M.F., Internal-External Control and beliefs about automobile travel. Journal of Research in Personality, 1973. 7(3): p. 288–293.

[15] Lajunen T. and Summala H., Driving experience, personality, and skill and safety-motive dimensions in drivers’ self-assessments. Personality and Individual Differences, 1995. 19(3): p. 307–318.

[16] Alper S. and Özkan T., Do internals speed less and externals speed more to cope with the death anxiety? Transportation Research Part F: Traffic Psychology and Behaviour, 2015. 32: p. 68–77.

[17] Huang J. and Ford J., Driving locus of control and driving behavior: Inducing change through driver training. Transportation Research Part F Traffic Psychology and Behaviour, 2012. 15: p. 358–368.

[18] Gidron Y., Gal R., and Desevilya H.S., Internal locus of control moderates the effects of road-hostility on recalled driving behavior. Transportation Research Part F: Traffic Psychology and Behaviour, 2003. 6(2): p. 109–116.

[19] Huang J.L. and Ford J.K., Driving locus of control and driving behaviors: Inducing change through driver training. Transportation Research Part F: Traffic Psychology and Behaviour, 2012. 15(3): p. 358–368.

[20] Beaton D.E., et al., Guidelines for the process of cross-cultural adaptation of self-report measures. Spine, 1976. 25(24): p. 3186–91. 10.1097/00007632-200012150-00014 11124735

[21] Youssef D., et al., Examining self-reported aberrant behavior among Lebanese drivers using the Driver Behavior Questionnaire (DBQ). 2022.

[22] Comrey A.L. and Lee H.B., A first course in factor analysis. 2013: Psychology press.

[23] Reason J., et al., Errors and violations on the roads: a real distinction? Ergonomics, 1990. 33(10–11): p. 1315–32. doi: 10.1080/00140139008925335 20073122

[24] Rea L.M. and Parker R.A., Designing and conducting survey research: A comprehensive guide. 2014: John Wiley & Sons.

[25] Cronbach L.J., Coefficient alpha and the internal structure of tests. Psychometrika, 1951. 16(3): p. 297–334.

[26] Koo T.K. and Li M.Y., A Guideline of Selecting and Reporting Intraclass Correlation Coefficients for Reliability Research. Journal of Chiropractic Medicine, 2016. 15(2): p. 155–163. doi: 10.1016/j.jcm.2016.02.012 27330520 PMC4913118

[27] Cicchetti D., Guidelines, Criteria, and Rules of Thumb for Evaluating Normed and Standardized Assessment Instrument in Psychology. Psychological Assessment, 1994. 6: p. 284–290.

[28] Lawshe C.H.J.P.p., A quantitative approach to content validity. 1975. 28(4): p. 563–575.

[29] LAWSHE C.H., A QUANTITATIVE APPROACH TO CONTENT VALIDITY1. Personnel Psychology, 1975. 28(4): p. 563–575.

[30] Byrne B.M., Structural equation modeling with EQS: Basic concepts, applications, and programming, 2nd ed, in Structural equation modeling with EQS: Basic concepts, applications, and programming, 2nd ed. 2006, Lawrence Erlbaum Associates Publishers: Mahwah, NJ, US. p. xii, 440–xii, 440.

[31] Sun L., Ma Y., and Hua L., Adaptation and validity of the traffic locus of control scale in Chinese drivers. Personality and Individual Differences, 2020. 159: p. 109886.

[32] Levenson H., Differentiating among internality, powerful others, and chance. Research with the locus of control construct, 1981. 1: p. 15–63.

[33] Guastello S.J. and Guastello D.D., The relation between the locus of control construct and involvement in traffic accidents. The Journal of Psychology: Interdisciplinary and Applied, 1986. 120(3): p. 293–297. doi: 10.1080/00223980.1986.10545255 3746718

[34] Iversen H. and Rundmo T., Personality, Risky Driving and Accident Involvement Among Norwegian Drivers. Personality and Individual Differences, 2002. 33: p. 1251–1263.

[35] Luszczynska A. and Schwarzer R., The Role of Self-Efficacy in Health Self-Regulation, in The adaptive self: Personal continuity and intentional self-development. 2005, Hogrefe & Huber Publishers: Ashland, OH, US. p. 137–152.

[36] Özkan T. and Lajunen T., A new addition to DBQ: Positive driver behaviours scale. Transportation Research Part F: Traffic Psychology and Behaviour, 2005. 8(4–5): p. 355–368.

[37] Sümer N., Personality and behavioral predictors of traffic accidents: testing a contextual mediated model. Accident Analysis & Prevention, 2003. 35(6): p. 949–964. doi: 10.1016/s0001-4575(02)00103-3 12971930

