# Supplementary material for: Exploring the association between the Arabic version of the Traffic Locus of Control, driving behavior, and road traffic crashes: A multidimensional approach
Source: PLoS One. 2024 May 23;19(5):e0303518. doi: 10.1371/journal.pone.0303518 (PMC11115203; doi:10.1371/journal.pone.0303518)
Supplement: S1 Questionnaire — (DOCX) [file pone.0303518.s001.docx]

### Questionnaire

**استبيان الدراسة حول سلوك السائقين في لبنان**

هذا الاستبيان هوجزء من مشروع بحث حول تقييم السائق لسلوكه وقدراته كما ومعرفة كيفية تفاعله مع المواقف المختلفة التي تصادفه أثناء القيادة ، وعلاقة ذلك بسماته الشخصية وتأثير ذلك على احتمالية إنخراطه في صدام مروري. يتكون هذا الاستبيان من خمسة أقسام تشمل معلومات ديمغرافية حول السائق ، سلوكياته وقدراته وكل ذلك بناءً على وجهة نظره الشخصية وتقييمه الذاتي. من يقوم بتعبئة هذا الاستبيان سوف يبقى مجهول الهوية ولن يطلب منه اي معلومات شخصية. ونشير الى أن المعلومات الواردة في هذا الاستبيان هي لغرض البحث العلمي وسيتم التعامل معها بسريّة عالية. يتطلب هذا الاستبيان 10-15 دقيقة لاكماله. كما ونشجع المشاركين في هذه الدراسة على الاجابة على الأسئلة بشكل موضوعي. مشاركتك في هذه الدراسة طوعية وستكون محل تقدير كبير. لمزيد من المعلومات يمكن الاتصال عبر البريد الالكتروني: dalalyoussef.ipnet@gmail.com

في حال موافقتك على المشاركة، الرجاء تعبئة الموافقة المستنيرة المرفقة ومن ثم القيام بتعبئة الإستبيان

| **القسم الأول : معلومات ديمغرافية** | | | | | | | | | | | | | |
| --- | --- | --- | --- | --- | --- | --- | --- | --- | --- | --- | --- | --- | --- |
| **1** | **العمر** | |  | | | | | | | | | | |
| **2** | **الجنس** | | 1. **ذكر** | | 1. **أنثى** | | | | | | | | |
| **3** | **الجنسية** | | 1. **لبناني** | 1. **سوري** | 1. **فلسطيني** | 1. غيره | | | | | | | |
| **4** | **الوظيفة** | | 1. **طالب** | 1. **أعمل كسائق** | 1. **أعمل ولكن ليس كسائق** | 1. عاطل عن العمل | | | | | | | |
| **5** | **الوضع الإجتماعي** | | 1. **اعزب** | 1. **متزوج** | 1. **أرمل** | 1. مطلق | | | | | | | |
| **6** | **المستوى التعليمي** | | 1. **لا أجيد القراءة أو الكتابة** | 1. **ابتدائي /متوسط** | 1. **ثانوي أو BT** | 1. جامعي أو TS | | 1. ماستر أو أكثر | | | | | |
| **7** | **عدد افراد الاسرة** | | **………………………………………………………………………………………….** | | | | | | | | | | |
| **8** | **الدخل الشهري للاسرة** | | 1. **اقل من مليون ليرة** | | 1. **1مليون مليون** | 1. **مليونين - ملايين** | | 1. **أكثر من 4 ملايين** | | | | | |
| **9** | **عدد السيارات في المنزل** | | 1. **لا يوجد** | 1. **1** | | 1. (2-3) | | 1. اكثر من 3 | | | | | |
| **10** | **رخصة قيادة** | | 1. **نعم** | | 1. **لا** | | | | | | | | |
| **11** | **نوع رخصة القيادة** | | 1. **خصوصي** | | 1. **عمومي** | | | | | | | | |
| **12** | **نوع المركبة** | | 1. **سيارة** | | 1. **فان/BUS** | 1. دراجة نارية | | 1. شاحنة | | | | | |
| **13** | **الاستعمال الأساسي للمركبة** | | 1. **الترفيه/ الزيارات الاجتماعية** | | 1. **الذهاب والعودة الى العمل** | 1. أعمل كسائق | | 1. لا أعمل | | | | | |
| **14** | **قوة المركبة** | | 1. **2 wheels** | | 1. **4 wheels** | | | | | | | | |
| **15** | **عدد سنوات الخبرة في القيادة** | |  | | | | | | | | | | |
| **16** | **عدد الكيلومترات المقطوعة سنويا** | | 1. **0-2000 km** | 1. **2000-4000 km** | 1. **4000-6000 km** | 1. **6000-8000 km** | | 1. **>8000 km** | | | | | |
| **18** | **صدامات مرورية في السنوات الثلاث الماضية** | | 1. **نعم** | | 1. **لا** | | | | | | | | |
| **19** | **عدد الصدامات مرورية في السنوات الثلاث الماضية** | | **……………………………………………………………………………………………** | | | | | | | | | | |
| **20** | **أنواع الصدامات المرورية(1)** | | 1. **أضرار مادية** | | 1. **إصابات جسدية** | | | 1. **وفاة** | | | | | |
| **20** | **أنواع الصدامات المرورية(2)** | | 1. **أضرار مادية** | | 1. **إصابات جسدية** | | | 1. **وفاة** | | | | | |
| **21** | **عدد المرات التي تم ضبطك من قبل شرطة السير في السنة الماضية** | |  | | | | | | | | | | |
| **22** | **أنواع المخالفات التي تم ضبطك** | | **1.  مخالفات السرعة** | | **2. الاشارات الضوئية** | | | **3**. حزام الامان | | | | | |
|  |  |  | 1. **مخالفات استخدام الهاتف اثناء القيادة** | | | 1. القياده (عكس السير) | | 1. الاصطفاف (الركن) الخاطئ | | | | | |
|  |  |  | 1. **مخالفات اخرى (يرجى التحديد)** | | | | | | | | | | |
| **القسم الثاني :سلوك السائق** | | | | | | | | | | | | | |
| يرجى الاجابة عن الاسئلة التالية استنادا الى افضل تقدير لديكم، يرجى اختيار **اجابة واحدة فقط عن كل سؤال** | | | | | | | | | | | | | |
|  | |  | | | | | **ابدا** | | **نادرا** | **احيانا** | **غالبا** | **معظم الاحيان** | **دائما** |
| **1** | | **تحاول الابتعاد والتملص من اشارات المرور** | | | | | 0 | | 1 | 2 | 3 | 4 | 5 |
| **2** | | **عند التحقق من عداد السرعة، اكتشفت انك تقود بسرعة أكبر من السرعة المسموح بها** | | | | | 0 | | 1 | 2 | 3 | 4 | 5 |
| **3** | | **تخرج من السيارة وتنسى المفاتيح في الداخل** | | | | | 0 | | 1 | 2 | 3 | 4 | 5 |
| **4** | | **تقود بنفس السرعة في الليل على الطرقات المعتمة كما على الطرقات المضاءة** | | | | | 0 | | 1 | 2 | 3 | 4 | 5 |
| **5** | | **تحاول الخروج بالمركبة من خط او من من موقف(صفة) دون اعطاء اشارة تحذيرية** | | | | | 0 | | 1 | 2 | 3 | 4 | 5 |
| **6** | | **تقترب كثيرا من المركبة التي أمامك او تشغل الضوء (Flashضويلا وطفيلا) كإشارة لحث السائق الذي أمامك على الإسراع أو افساح الطريق** | | | | | 0 | | 1 | 2 | 3 | 4 | 5 |
| **7** | | **تنسى أين ركنت مركبتك في موقف للسيارات(Parking)** | | | | | 0 | | 1 | 2 | 3 | 4 | 5 |
| **8** | | **بسبب التلهي أو الإنشغال، أدركت متأخرا أن المركبة التي أمامك تباطأت وأن عليّك الدوس على الفرامل لتجنب الصدام(الحادث)** | | | | | 0 | | 1 | 2 | 3 | 4 | 5 |
| **9** | | **كنت تنوي تشغيل ماسحات الزجاج الأمامي(المساحات) ، ولكنك قمت بتشغيل الأنوار بدلاً من ذلك ، أو العكس** | | | | | 0 | | 1 | 2 | 3 | 4 | 5 |
| **10** | | **انعطفت نحو طريق رئيسي ذو حركة سير معاكسة وتفاجأت بمركبة (سيارة، دراجة نارية....) قادمة حيُث أنك لم ترها أو أخطأت في تقدير السرعة** | | | | | 0 | | 1 | 2 | 3 | 4 | 5 |
| **11** | | **أسأت تقدير المساحة اللازمة لركن المركبة في موقف/على الطريق واصطدمت (ضربت) بالسيارة المجاورة.** | | | | | 0 | | 1 | 2 | 3 | 4 | 5 |
| **12** | | **لا تتذكر بشكل واضح الطريق التي سلكتها للتوّ** | | | | | 0 | | 1 | 2 | 3 | 4 | 5 |
| **13** | | **فاتك المخرج (المكان المخصص للخروج من الطريق ) وعليّك أن تقوم بجولة (لفّة) طويلة للعودة** | | | | | 0 | | 1 | 2 | 3 | 4 | 5 |
| **14** | | **وأنت تقود المركبة نسيت الغيار الذي تستعمله وعليك التأكد من ذلك بيدك** | | | | | 0 | | 1 | 2 | 3 | 4 | 5 |
| **15** | | **عالق وراء مركبة تسير ببطء على طريق سريع يدفعك انزعاجك لمحاولة تجاوزها إما من الجهة الخاطئة أو من الجهة الصحيحة على الرغم من خطورة ذلك** | | | | | 0 | | 1 | 2 | 3 | 4 | 5 |
| **16** | | **تريد الذهاب المكان (أ) فتنتبه انك تقود المركبة على الطريق الخاطئ (ب) لكونه الطريق الذي تسلكه بالعادة** | | | | | 0 | | 1 | 2 | 3 | 4 | 5 |
| **17** | | **تجازف بالمرور والاشارة تحولت الى اللون الأحمر** | | | | | 0 | | 1 | 2 | 3 | 4 | 5 |
| **18** | | **اغضبك تصرف أحد السائقين،تشتمه أوتستخدم الزمور أو تقوم بحركة بذيئة أو تلاحقته لتوبيخه و للتعبير عن انزعاجك** | | | | | 0 | | 1 | 2 | 3 | 4 | 5 |
| **19** | | **لديك نفورًا من فئة معينة من مستخدمي الطريق ، تحاول ابراز امتعاضك وعداؤك بأي وسيلة يمكنك من خلالها ذلك** | | | | | 0 | | 1 | 2 | 3 | 4 | 5 |
| **20** | | **حاولت التجاوز بدون النظر (تفحّص) في المرايا فكادت أن تصصدم بك سيارة من الخلف حيث أنها كانت قد باشرت بالتجاوز قبلك** | | | | | 0 | | 1 | 2 | 3 | 4 | 5 |
| **21** | | **تتجاهل عمدا السرعة المحددة على الطريق اذا كنت تقود في وقت متأخر من الليل أو في وقت باكر في الصباح أو في غياب شرطي** | | | | | 0 | | 1 | 2 | 3 | 4 | 5 |
| **22** | | **تنسَى تاريخ انتهاء صلاحية التأمين او رسوم الميكانيك أو صلاحية رخصة السوق(أوراق السيارة)....وتكتشف أنك تقود سيارتك بطريقة غير قانونية** | | | | | 0 | | 1 | 2 | 3 | 4 | 5 |
| **23** | | **نسيت المصابيح الأمامية (مضاءة) الى أن قام أحد السائقين بتنبيهك ب (FLASH)** | | | | | 0 | | 1 | 2 | 3 | 4 | 5 |
| **24** | | **تنتبه جيدًا لحركة المرور اذا كان هناك خط من المركبات التي تنعطف إلى الطريق الرئيسي حيث أنت موجود** | | | | | 0 | | 1 | 2 | 3 | 4 | 5 |
| **25** | | **عند العودة من حفلة أو مطعم أو PUB، تقود المركبة على الرغم من أنك تدرك أنه من الممكن انك تجاوزت الحد القانوني لنسبة الكحول الدم** | | | | | 0 | | 1 | 2 | 3 | 4 | 5 |
| **26** | | **وانت غارق في أفكارك وانتباهك مشتت(تلهي)، لم تلاحظ وجود شخص ينتظر العبور عند ممر المشاة أوشخص يخرج من وراء حافلة أو يريد الذهاب إلى شارع جانبي من طريق رئيسي** | | | | | 0 | | 1 | 2 | 3 | 4 | 5 |
| **27** | | **تركن (توقف السيارة) في مكان ممنوع الوقوف على الرغم من خطر الغرامة** | | | | | 0 | | 1 | 2 | 3 | 4 | 5 |
| **28** | | **تخطئ في تقدير سرعة للمركبة القادمة باتجاهي عند التجاوز** | | | | | 0 | | 1 | 2 | 3 | 4 | 5 |
| **29** | | **اصطدمت بشيء عند الرجوع الى الخلف لم يسبق لك رؤيته أو التنبه له** | | | | | 0 | | 1 | 2 | 3 | 4 | 5 |
| **30** | | **لم تقم بتخطيط مسارك بشكل جيد ، فواجهت ازدحام المروري كان من الممكن أن تتفاداه** | | | | | 0 | | 1 | 2 | 3 | 4 | 5 |
| **31** | | **تجاوزت خط واحد من المركبات التي تتحرك ببطء ، فقط لتكتشف أنها تنتظر لتعبر من فتحة (حارة) واحدة أوبسبب أشغال** | | | | | 0 | | 1 | 2 | 3 | 4 | 5 |
| **32** | | **بتقص الكوع" اي تقطع الزاوية على المنعطف الأيمن وتضطر إلى اللف " الانحناء (اللف) بعنف لتفادي مركبة قادمة** | | | | | 0 | | 1 | 2 | 3 | 4 | 5 |
| **33** | | **دخلت في الطريق الخطأ عند المستديرة (ألدوار) أو عند الاقتراب من تقاطع طريق** | | | | | 0 | | 1 | 2 | 3 | 4 | 5 |
| **34** | | **فشلت في قراءة الارشادات (الاشارات) بشكل صحيح ، خرجت من المستديرة الى الطريق الخطأ** | | | | | 0 | | 1 | 2 | 3 | 4 | 5 |
| **35** | | **لم تنتبه لمركبة(Bus, Van,سيارة ) اعطت اشارة بنيتها بالخروج من "الصفة" اوتغيير الخط** | | | | | 0 | | 1 | 2 | 3 | 4 | 5 |
| **36** | | **تجاهلت اشارة „إفسح الطريق‟ ، وأصبح عليك أن تتجنب بصعوبة الاصطدام بالمركبات المتجهة في الطريق الصحيح** | | | | | 0 | | 1 | 2 | 3 | 4 | 5 |
| **37** | | **. قبل الانسحاب ، وتغيير الممرات والانعطاف.... في التحقق من المرآة فشلت** | | | | | 0 | | 1 | 2 | 3 | 4 | 5 |
| **38** | | **حاولت تجاوز مركبة لم تكن قد لاحظتها تشير إلى عزمها على التوجه إلى اليمين** | | | | | 0 | | 1 | 2 | 3 | 4 | 5 |
| **39** | | **تتعمد السير في الطريق الخطأ في شارع في اتجاه واحدا ولكنه قليل الحركة** | | | | | 0 | | 1 | 2 | 3 | 4 | 5 |
| **40** | | **تتجاهل الأضواء الحمراء والصفراء عند القيادة في ساعة متأخرة من الليل** | | | | | 0 | | 1 | 2 | 3 | 4 | 5 |
| **41** | | **تقوم أثناء القيادة بالتلهي بتغيير قناة راديو ، المكيف، استعمال الهاتف....إلخ.** | | | | | 0 | | 1 | 2 | 3 | 4 | 5 |
| **42** | | **تنخرط(تدخل) في سباقات غير رسمية مع السائقين الآخرين** | | | | | 0 | | 1 | 2 | 3 | 4 | 5 |
| **43** | | **تسابق السيارات القادمة على طريق ضيق باتجاه فتحة صغيرة (مخرج)** | | | | | 0 | | 1 | 2 | 3 | 4 | 5 |
| **44** | | **تدوس على الفرامل بقوة على طريق زلق أو توجه بطريقة خاطئة في طريق منزلق** | | | | | 0 | | 1 | 2 | 3 | 4 | 5 |
| **45** | | **تسيء تقدير الوقت اللازم للعبور الخاص بي عندما تنعطف وبصعوبة تتجنب الصدام.** | | | | | 0 | | 1 | 2 | 3 | 4 | 5 |

| **القسم الثالث : أسباب الصدامات** | | | | | | |
| --- | --- | --- | --- | --- | --- | --- |
| **في هذا المقياس ، ستجد قائمة بالأسباب المحتملة للحوادث. يرجى الإشارة إلى أي مدى كان من الممكن أن تكون هذه الأسباب الستة عشر قد تسببت أو ستتسبب في وقوع حادث وذلك وفقاَ أسلوب وظروف القيادة الخاصة بك.** | | | | | | |
| **#** |  | **غير ممكن أبدا** | **غير ممكن** | **ليس ممكن وليس غير ممكن** | **ممكن** | **ممكن للغاية** |
| **1** | **وقوع حادث سيارة أم لا : يرتبط غالباً بأوجه القصور في مهاراتي في القيادة** | **1** | **2** | **3** | **4** | **5** |
| **2** | **وقوع حادث سيارة أو لا :يرتبط غالباً بالمخاطرة التي أقوم بها أثناء القيادة** | **1** | **2** | **3** | **4** | **5** |
| **3** | **وقوع حادث سيارة أم لا : يرتبط غالباً بأوجه القصور في مهارات القيادة لدى السائقين الآخرين** | **1** | **2** | **3** | **4** | **5** |
| **4** | **وقوع حادث سيارة أو لا : يرتبط غالباً بالمخاطرة التي يقوم بها السائقين الآخرين أثناء القيادة** | **1** | **2** | **3** | **4** | **5** |
| **5** | **وقوع حادث سيارة أو لا : يرتبط غالباً بالحظ السيء** | **1** | **2** | **3** | **4** | **5** |
| **6** | **وقوع حادث سيارة أو لا : يرتبط غالباً بوجود طرقات خطرة** | **1** | **2** | **3** | **4** | **5** |
| **7** | **وقوع حادث سيارة أو لا : يرتبط غالباً بما إذا كنت أقود السيارة بسرعة عالية جدًا** | **1** | **2** | **3** | **4** | **5** |
| **8** | **وقوع حادث سيارة أو لا: يرتبط غالباً بما إذا كان السائقون الآخرون يقودون سرعة عالية جدًا** | **1** | **2** | **3** | **4** | **5** |
| **9** | **وقوع حادث سيارة أو لا: يرتبط غالباً بمّا اذا كنت أقود سيارتي بشكل متاخم (قريب جدا) من السيارة التي أمامي** | **1** | **2** | **3** | **4** | **5** |
| **10** | **وقوع حادث سيارة أو لا: يرتبط غالباً بما إذا كان السائقين الآخرون يقودون سياراتهم بشكل متاخم (قريب جدا) من سيارتي** | **1** | **2** | **3** | **4** | **5** |
| **11** | **وقوع حادث سيارة أو لا: يرتبط غالباً بالقضاء والقدر** | **1** | **2** | **3** | **4** | **5** |
| **12** | **. وقوع حادث سيارة أو لا: يرتبط بسوء الأحوال الجوية أو ظروف الإضاءة** | **1** | **2** | **3** | **4** | **5** |
| **13** | **وقوع حادث سيارة أو لا: يرتبط بحصول عطل ميكانيكي في السيارة** | **1** | **2** | **3** | **4** | **5** |
| **14** | **وقوع حادث سيارة أو لا: يرتبط غالبا بسائقين آخرين يقودون تحت تأثير الكحول** | **1** | **2** | **3** | **4** | **5** |
| **15** | **وقوع حادث سيارة أو لا: يرتبط غالبا بالتجاوز الخطير الذي يقوم به السائقون الآخرون** | **1** | **2** | **3** | **4** | **5** |
| **16** | **وقوع حادث سيارة أو لا: يرتبط غالبا بالتجاوز الخطير الذي أقوم به** | **1** | **2** | **3** | **4** | **5** |
